# Supplementary material for: Abscisic Acid and Its Receptors LANCL1 and LANCL2 Control Cardiomyocyte Mitochondrial Function, Expression of Contractile, Cytoskeletal and Ion Channel Proteins and Cell Proliferation via ERRα
Source: Antioxidants (Basel). 2023 Aug 30;12(9):1692. doi: 10.3390/antiox12091692 (PMC10526111; doi:10.3390/antiox12091692)
Supplement: Supplementary file 1 [file antioxidants-12-01692-s001.zip › antioxidants-2557746-supplementary.pdf]

antioxidants

MDPI

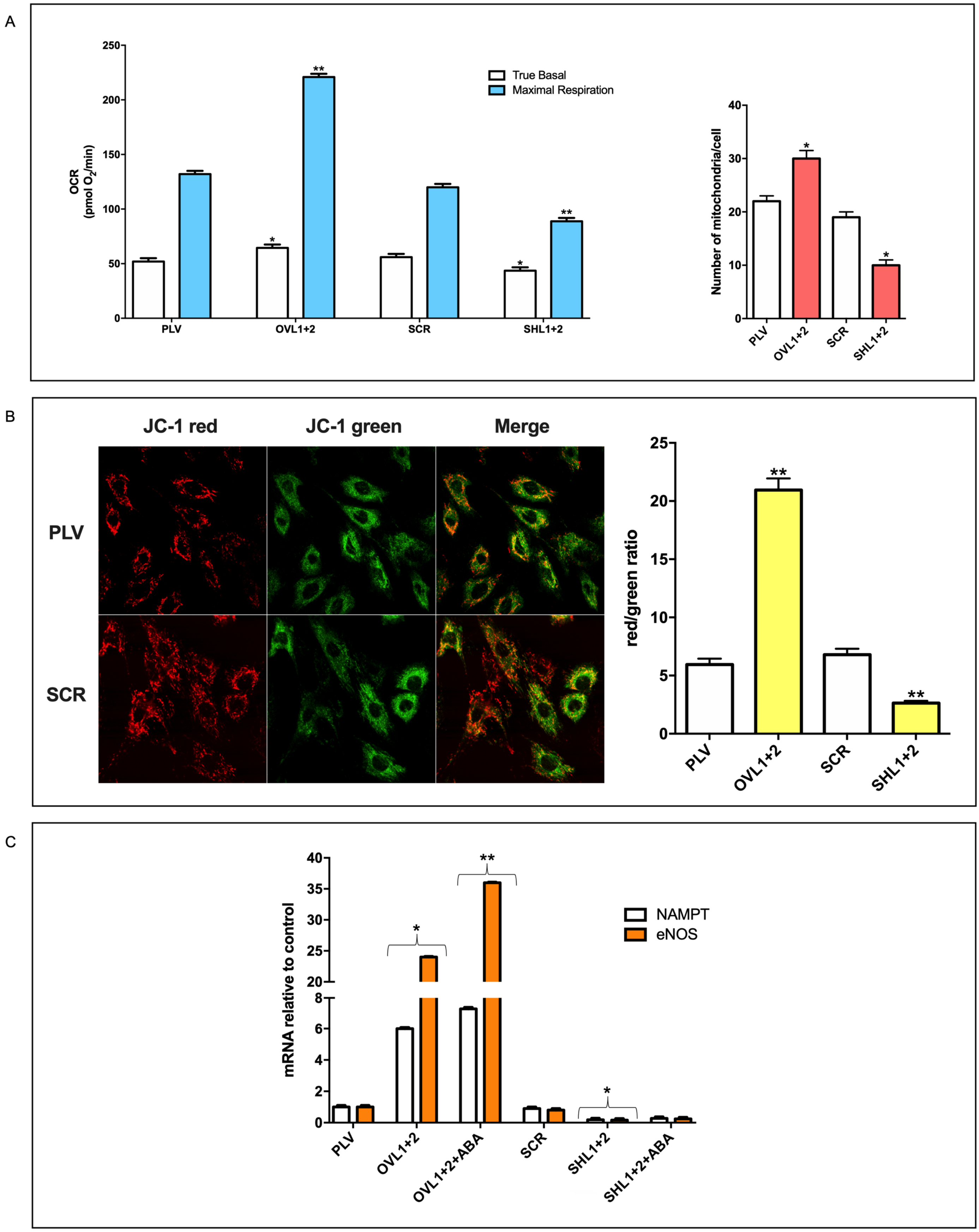

Figure S1. The vectors used to overexpress or to silence LANCL1 and LANCL2 do not affect mitochondrial number, respiration and proton gradient and NAMPT and eNOS transcription in H9c2.

Panel A. Left panel, basal and maximal respiration rates of H9c2 infected with the empty vector (PLV) used for LANCL1/2 overexpression (OVL1+2), or with a vector containing the scrambled sequences (SCR) for LANCL1/2 silencing (SHL1+2). Right panel, mitochondrial number/cell estimated by means of Mitotracker fluorescence in the same cell types. Mean ± SD of three determinations. *p<0.05 and **p<0.01 OVL1+2 vs SHL1+2 by unpaired t-test.

Panel B. Left panel, representative images of PLV and SCR H9c2 cells loaded with the potentiometric dye JC-1. Right panel, red/green fluorescence ratio calculated in 4 microscopic fields (scale bar: 20μm). The bars of LANCL1/2-overexpressing and double silenced cells (yellow) have been added for comparison and are the same values shown in Figure 1E. **p<0.01 OVL1+2 vs. SHL1+2 by unpaired t-test.

Panel C. mRNA levels of the indicated genes measured by qPCR are expressed relative to PLV cells. OVL1+2 and SHL1+2 cells were incubated in the absence or in the presence of 100 nM ABA for 4 hours (as in Figure 2). Results shown are the mean ± SD of 3 experiments. *p<0.01 relative to the respective control untreated cells (PLV or SCR) and **p<0.05 relative to OVL1+2 cells by unpaired t-test.

A
B

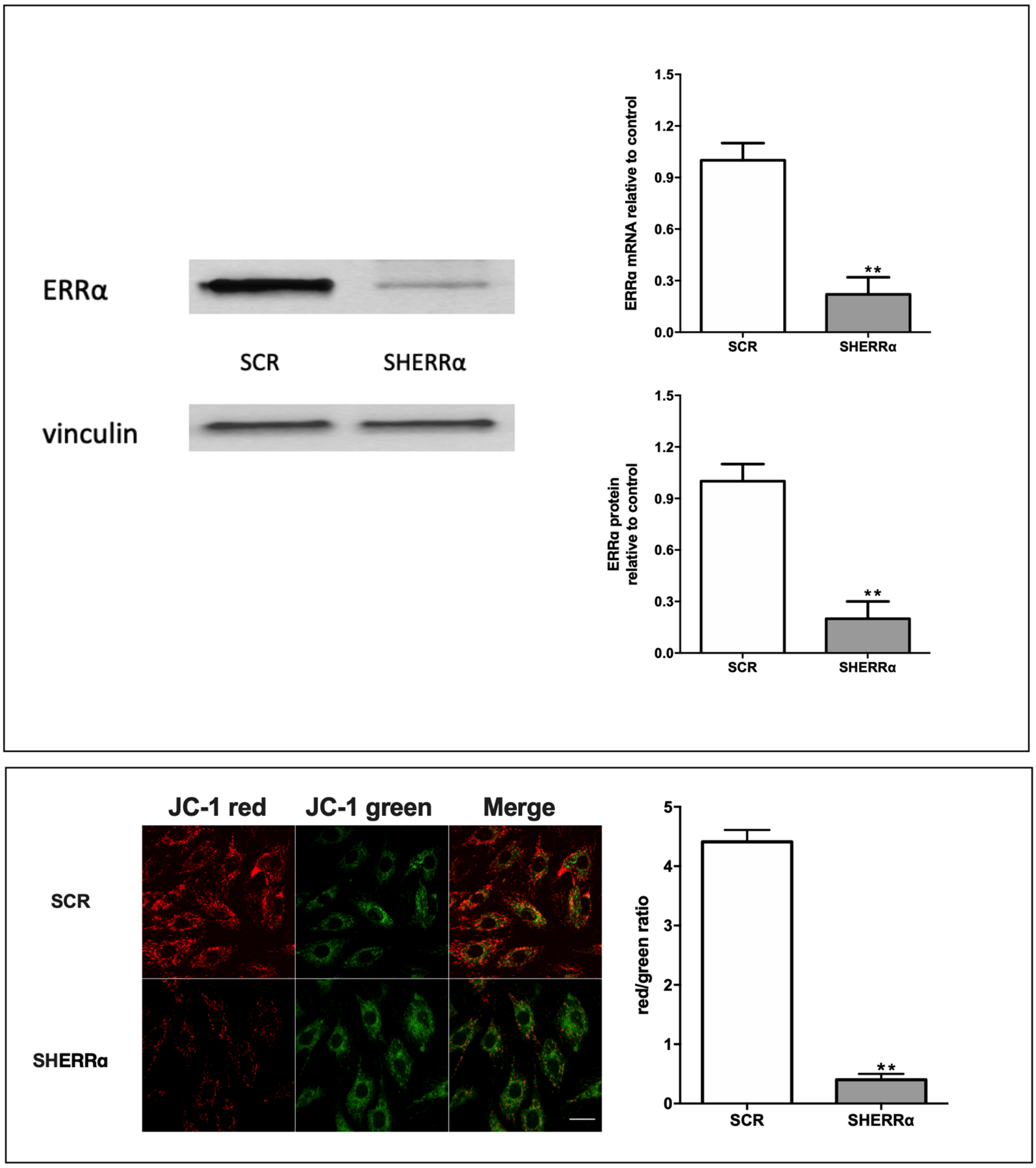

Figure S2. ERRα silencing reduces mitochondrial proton gradient in wild-type H9c2 cardiomyocytes. (A) Left panel, representative Western blots of ERRα protein in in wild-type H9c2 (untransfected for LANCL overexpression) silenced for ERRα, compared with controls, transfected with the scrambled sequences (SCR); upper right panel, ERRα mRNA levels in SHERRα cells relative to SCR; lower right panel, densitometric quantitation of the ERRα protein in SHERRα cells relative to SCR. Values are normalized on vinculin. **p<0.01 relative to SCR control cells by unpaired t-test. Data shown are the mean ± SD of 3 experiments per group, with each value calculated in triplicate. (B) SHERRα cells were loaded with the $\Delta\Psi$-sensitive ratiometric fluorescent dye JC-1, a higher $\Delta\Psi$ resulting in a higher red/green fluorescence ratio. Left panel, representative confocal microscopy images; right panel, red/green ratio calculated in at least 3 microscopic fields (scale bar: 20µm) for each experiment. **p<0.01 relative to SCR cells by unpaired t-test.

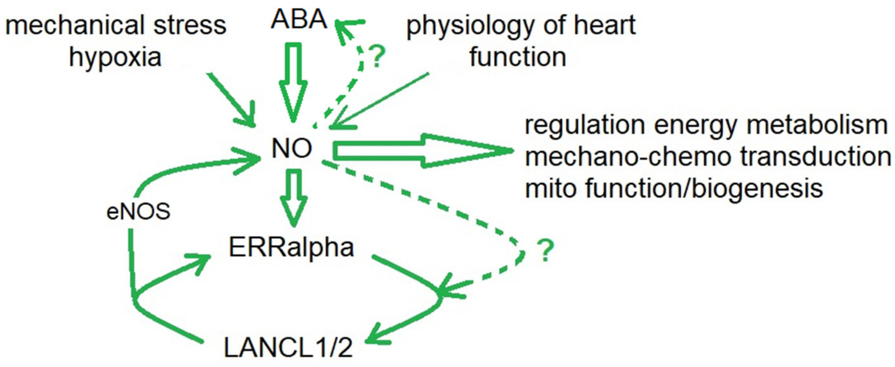

Figure S3. What triggers ERRα and LANCL1/2 activation in the "stressed" heart? Nitric oxide (NO), locally produced in the heart not only by cardiomyocytes but also by endothelial cells and erythrocytes, plays a central role in allowing adaptation of cardiomyocyte metabolism, contraction and electrical conduction to changing physiological conditions [2,61-64]. Here we observed that LANCL1/2 and ERRα are linked in a feed-forward mechanism of reciprocal transcriptional activation (Figure 5A). In addition, both ERRα [64] and LANCL1/2 [5] activate NO-generating eNOS. Here, we also show that NO is required for LANCL1/2-induced stimulation of ERRα transcription in H9c2 cells (Figure 5A). Finally, ABA release is stimulated by hypoxia in H9c2 and ABA in turn stimulates NO production and LANCL1/2-expression [5]. Does NO activate ABA release from H9c2 (dotted line)? Is NO necessary for ERRα-mediated LANCL1/2 transcriptional activation (dotted line)? These questions are still awaiting an answer. In any case, NO and ABA, via LANCL1/2 and ERRα mediate all transcriptional and functional responses of H9c2 described in this study.

Table S1. Primer sequences used to amplify rat target genes.

Rat genes	Accession N.	Forward Primer 5′-3′	Reverse Primer 5′-3′
Hprt1	NM_012583	TTGGTCAAGCAGTACAGCCC	TGGCCTGTATCCAACACTTCG
Lancl1	NM_053723	TCTTGCTCCTCATCCTGCTCATC	CACTGTACTCGCCGAAGGTCTC
Lancl2	NM_001014187	GGTGCCACGGTGCTCCAG	CCTCGCTGCCAAATCACATCAC
Slc2a1	NM_138827	GACCCTGCACCTCATTGGT	CTCAGATAGGACATCCAGGGC
Slc2a4	NM_012751	CCAGCCTACCGCCACCATAG	TTCCAGCAGCAGCAGAGC
Pfk1	NM_031715	AGTTGGTATCTTCACGGGCG	CATAGACACGCTCTCCCACG
PK	M24359	CCAAGAGAACGAGCTACCCC	TGGAGCCCCACTTAAAGCAG
Gapdh	AF106860	ATGACTCTACCCACGGCAAG	CTGGAAGATGGTGATGGGTT
Pdha1	NM_001004072	GATGGAGCTAAAGGCGGATCA	TCCGTAGGGTTTATGCCAGC

Ucp3	NM_013167	CCCCCTACACTGTATGCTGA	TTCCAGGATCCCAGACGCA
Mt-nd1	KJ530565	CCACGCTTCCGTTACGATCA	GTATGGTGGTACTCCCGCTG
ANT1	D12770	TGGATGATTGCGCAGAGTGT	AATATCAGCCCCTTTCCGGC
Cpt1b	NM_013200	TGTCTACCTCCGAAGCAGGA	TGAACGGCATTGCCTAGACG
Acads	NM_022512	GAGAAGGAGTTGGTCCCCATT	CCGAGCTCACCCATCTTCTTA
Esrra	NM_001008511	CCCTGACAGTCCAAAGGGTT	CATCCTCCTCCTCCTTGTGC
KCNK2	AF385402	CAGGTGGGTCGGACATTGAA	CCCGTAGCCAGTCTCCAATC
Cacna1c	NM_012517	CTGCCCTATGTGGCCCTTTT	TCTGTGGTGTCATTCAGGGC
Scn1b	NM_001271045	CTGCTGGCTCTCGTGGTG	CCATACACTGCCTCGGTCTC
Ccnd1	NM_171992	CTACCGCACAACGCACTTTC	CAGGCTTGACTCCAGAAGGG
Ccnd2	NM_022267	CCAAGATCACCCACACCGAT	TTGTGCTGCTCTTGACGGAA
Ccnd3	NM_012766	AACCACGCCCCTGACTATTG	CACTTGAGCTTCCCCAGGAC
Ccne1	NM_001100821	GACAAGACTGTGAAAAGCCAGG	GATGAAAGAGCAGGGGTCCA
Ccna2	NM_053702	CTCTTTACCCGGAGCCAGAAA	ACATTCACTGGCTTTTCGTCTT
Cdk2	NM_199501	GGCTGCATCTTTGCCGAAAT	CTGGCCAAACCACCTCATCT
cdk4	L11007	GTACAAAGCCCGAGATCCCC	ACCTCACGAACTGTGCTGAC
E2f4	NM_001271345	TTGAGCCCATCAAGGCAGAC	CGGAGCTCATGCACTCTCTT
Actc1	NM_019183	GAGCTGTCTTCCCGTCCATC	TTGCTCTGGGCTTCATCACC

Tubb2a	NM_001109119	ACTTGCAGCTGGAGAGGATCA	CACTAGGATGGCCCGAGGTA
Ctnnb1	NM_053357	TACGAGCACATCAGGACACC	TGGAGAGCTCCAGTACACCC
Myh7	NM_017240	CAGCAGTTGGATGAGCGACT	GCTCATCCTCAATCCTGGCAT
Gja1	NM_012567	TTACAACAAGCAAGCCAGCG	GGGAGTTGGAGATGGTGCTT
Prkaa2	NM_019142	AGAAGCAGAAGCACGACGG	GAAGGTGCCGACGCCC
Ppargc1a	NM_031347	GCACACATCGCAATTCTCCC	CTCTGCGGTATTCGTCCCTC
Sirt1	NM_001372090	CAGTGTCATGGTTCCTTTGC	CACCGAGGAACTACCTGAT
Nampt	NM_177928	TCGGTTCTGGTGGAGGTTTGCTAC	TCCCTGCTGGCGTCCTATGTAAAG
Nos3	NM_021838	AGGCCTTGGTATTGGTGGTG	TAGGGGCCCGACATTTCCAT
Fgf21	NM_130752	CACACCGCAGTCCAGAAAGT	CCTAGAGGCTTTGACACCCA

Table S2. Primary and secondary antibodies used for Western blot.

**Primary Antibody**	**Host**	**Concentrations**	**Manufacturer**
Anti-LANCL1	Rabbit	1:250	Novus Biologicals
Anti-LANCL2	Mouse	1:1000	Reference [65]
Anti-ERRα	Mouse	1:200	Santa Cruz Biotechnology Inc., California
Anti-vinculin	Rabbit	1:1000	Cell Signaling Technology, Danvers, MA
**Secondary Antibody**	**Concentrations**		**Manufacturer**
Anti-Mouse	1:2000		Santa Cruz Biotechnology Inc., California

Anti-Rabbit	1:1000	Santa Cruz Biotechnology Inc., California
